# A Droplet Evaporimeter for Evaluating Eye Care Formulations

**DOI:** 10.1167/tvst.15.3.7

**Published:** 2026-03-09

**Authors:** Cheng-Chun Peng, Ying Zheng, Xinfeng Charlie Shi, George Yao, Clayton J. Radke, James Yuliang Wu

**Affiliations:** 1 Alcon Research LLC, Fort Worth, TX, & Johns Creek, GA, USA; 2 Chemical and Biomolecular Engineering Department, University of California, Berkeley, CA, USA; 3 Herbert Wertheim Scholl of Optometry & Vision Science, University of California, Berkeley, CA, USA

**Keywords:** tear evaporation, dry eye, eye drop, evaporimeter, emulsion

## Abstract

**Purpose:**

This study explores the development and application of a convenient droplet-based evaporimeter system as a research tool to investigate the evaporation of lubricating eye drop formulations and potentially other ophthalmic applications.

**Method:**

The evaporimeter integrates a goniometer with an environmental control system, allowing for precise test solution adjustment and a wide range of environmental settings. To verify the system design, pure water evaporation was conducted under various environmental conditions. Measured water evaporation rates aligned with the theoretical kinetic mass-transfer model. Evaporation evaluations were performed under conditions simulating the ocular surface in typical indoor environments. This study compared the evaporative flux of three baseline solutions and various commercial lubricating eye drop formulations.

**Results:**

Phosphate-buffered saline (PBS) had an evaporative flux similar to water, whereas simulated artificial tear solution (ATS), containing tear components, exhibited a lower evaporative flux. Among the tested eye drops, Systane Pro Preservative Free demonstrated the greatest reduction in evaporative flux, highlighting a potential synergistic effect between mineral oil and hyaluronic acid.

**Conclusions:**

An advanced evaporation evaluation system designed for in vitro and ex vivo research has been established to provide a rapid method with tunable sample/environment conditions to assess evaporative flux from eye drop formulations. This study enables future work to investigate eye drop formulations and their interactions with tear components to better understand their effects on reducing tear evaporation in dry eye.

**Translational Relevance:**

This work provides a reliable method to evaluate the effectiveness of eye drop formulations in reducing tear evaporation, which is essential for managing dry eye.

## Introduction

Tear evaporation is crucial in ophthalmology and optometry because it directly impacts the health and stability of the tear film, which is essential for maintaining clear vision and overall eye comfort.^1^ A general view of the tear film consists of two to three layers: the separated water-insoluble lipid layer and the aqueous and mucin layer(s), which are viewed as two distinguished layers or one continuous phase.^2^ A healthy tear film lipid layer, produced by the meibomian glands, spreads over the aqueous layer to prevent it from evaporating too quickly.^1^^,^^3^ Rapid tear evaporation increases tear osmolarity and destabilizes the tear film, leading to dry eye symptoms and eventually triggering a cycle of inflammation and tissue damage on the ocular surface. This cycle can perpetuate dry eye symptoms and make the condition more difficult to manage.^4^ By addressing tear evaporation, ophthalmologists and optometrists can better manage dry eye disease, improve patient comfort, and protect the health of the ocular surface. Understanding tear evaporation helps eye care professionals develop effective treatment plans. This may include therapies to improve meibomian gland function, use of lubricating eye drops, and lifestyle changes to reduce environmental factors that contribute to tear evaporation.^5^

Multiple instruments have been developed to measure tear evaporation using various methods, as documented in well-reviewed publications, including both in vivo and in vitro devices.^6^^,^^7^ The most common approach involves an evaporimeter that measures the mass difference due to evaporation by detecting relative humidity change or water mass loss/gain on the donor/receiving side vessels. Environmental conditions in these systems are typically described as an open or closed chamber with or without ventilation. Additionally, several indirect approaches, such as infrared thermography to monitor ocular surface temperature^8^^–^^10^ and spectral interferometry to measure thinning of the tear film due to tear evaporation,^11^^–^^13^ can convert the results to tear evaporation rate. Most of these approaches are designed primarily for in vivo on-eye tear evaporation measurement.

For lubricating eye drop development, the most common method to evaluate evaporation, sometimes referred to as the opposite of “moisture retention,” is gravimetric vapor sorption.^14^ This technique involves exposing a solution sample to a controlled environment with set temperature and humidity and measuring the sample's mass change over time. The required test sample size depends on the detection limit of mass change from the analyzer, making this method suitable for in vitro studies, where the amount of solution sample is not a major concern. However, it becomes challenging when investigating ex vivo tear samples and their components, which are typically available in volumes of only a few microliters or nanograms in mass.

In recent years, several researchers have focused on developing droplet-based evaporimeters^15^^–^^18^ to measure evaporation through drop shape image analysis that requires relatively small quantities of sample, adapting from the common droplet-based tensiometry techniques. A droplet of the test solution is formed in a controlled environment with set temperature and relative humidity, and the drop volume and surface area are continuously monitored to ascertain evaporation loss. The major advantage of using droplet-based evaporimetry is the small sample solution size required for each measurement, typically around 30 µL. Accordingly, it is possible to study ex vivo tear samples from individual human subjects.^15^^,^^19^ Additionally, it is possible to spread water-insoluble meibum/lipids over the formed droplet, creating a two-layer droplet that better mimics the tear film structure.^15^^,^^17^

In this study, we develop a modified droplet-based evaporimeter that allows for fine adjustment for test solution volume and various dispensing/dosing methods and offers a wide range of environmental conditions for evaporation measurement. The feasibility of the evaporation system is confirmed by measuring pure water evaporation under various conditions, in accordance with a physical evaporation model based on mass-transfer kinetics. The apparatus is primarily designed for in vitro and ex vivo research. The new instrument provides a fast and flexible method to measure evaporation from lubricating eye drop formulations for development screening purposes. In the future, the droplet technique can investigate the role of tear components on evaporation rates.

## Materials and Methods

### Evaporimeter System

**Figure 1.**
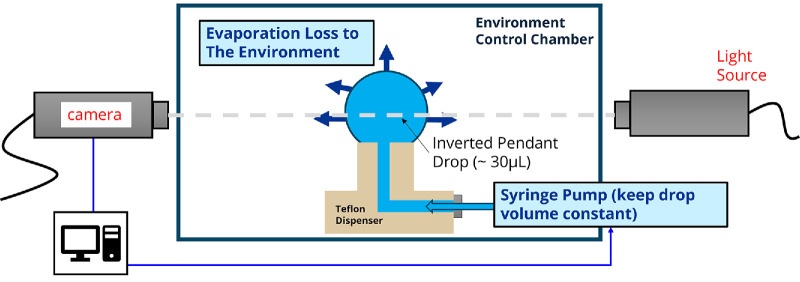
Schematic of droplet evaporimeter.

As illustrated in Figure 1, the customized evaporimeter system is based on drop shape analysis and consists of a goniometer with a dispensing syringe pump (model 590-U4; Ramé-Hart, Succasunna, NJ, USA) and accompanying controller software (DropImage, version 3.23.05.0). The dispensing capillary terminates in a closed chamber that surrounds the pinned sessile droplet. Temperature and humidity are controlled (model 100-26-TH; Ramé-Hart), allowing the environment to be set at desired levels.

### Materials

Corning (Corning, NY, USA) phosphate-buffered saline (PBS, 1× without calcium and magnesium, pH 7.4 ± 0.1) was purchased from Mediatech (Manassas, VA, USA). Purified water with a resistivity of 18.2 MΩ-cm at 25°C was obtained using a Milli-Q Type 1 ultrapure water system from Millipore Sigma (Burlington, MA, USA). Simulated artificial tear solution (ATS) was prepared in-house following protocols previously described.^20^

Lubricating eye drop formulations include Systane Complete, Systane Hydration, and Systane Pro Preservative Free (PF) from Alcon (Fort Worth, TX, USA). Systane Complete is a commercial lubricating eye drop containing mineral oil, whereas Systane Hydration contains hyaluronic acid. The new Systane Pro PF formulation contains both mineral oil and hyaluronic acid. Additionally, four commercial eye drop formulations containing lipids and/or hyaluronic acid were tested: Blink Triple Care from Johnson & Johnson (Irvine, CA, USA), Théa IVIZIA from Similasan (Highlands Ranch, CO, USA), and Refresh Optive Advance and Refresh Optive Mega-3 from Allergan (Madison, NJ, USA). All eye drop formulations are commercially available over-the-counter products obtained from retail sources and were tested as received without modification.

### Evaporation Measurement

Environmental conditions were stabilized at the target temperature and relative humidity for at least 20 minutes prior to measurement to ensure equilibrium within the closed environment chamber. For each experimental run, a fresh 30 ± 1 µL drop of sample solution was syringe-injected through the custom-made Teflon dispenser adaptor portrayed in Figure 1. The in situ image of the symmetrically pinned sessile liquid drop was continuously analyzed to determine the real-time drop surface area and volume, as well as the surface tension of the liquid sample. Evaporation measurements were conducted in the constant volume mode, controlled by commercial system controller software, to maintain a consistent drop size by compensating for evaporation losses through the addition of the test sample solution. The syringe pump's volume was recorded simultaneously to monitor the volume loss. The volumetric evaporation rate for each run was calculated as the average rate of volume loss from the syringe pump over a 10-minute period, unless otherwise specified. Volumetric evaporative flux was then determined by dividing the volumetric evaporation rate by the average surface area of the drop.

The selected droplet volume of 30 µL reflects the typical drop size dispensed from commercial eye drop bottles, rather than the physiological human tear volume (∼7 µL). Importantly, evaporation occurs at the liquid–vapor interface and is governed by the surface area of that interface, not the liquid volume. Once expressed as evaporation flux (rate per unit area), the measurement becomes independent of droplet size or geometry. This principle underlies our approach and aligns with standard in vitro practice aimed at characterizing intrinsic properties rather than replicating tear film geometry.

We note that the solute concentration of the aqueous-based solutions is not perfectly constant during the evaporation measurement. As pure water evaporates, it is replaced by an equal volume of fresh solution at the original concentration to maintain the droplet volume, leading to a gradual increase in concentration within the measured drop. To minimize this effect, measurements are limited to the first 10 minutes after dispensing a fresh droplet. All pinned droplets exhibited less than a 12% volume-average increase in concentration from the original drop over 10 minutes. Empirical data indicate that the evaporation rate does not significantly change within the first 10 minutes for all tested samples. The average evaporation rate was determined by the slope of a linear fit between the volume loss from the syringe pump over 10 minutes, with an *R*^2^ value greater than 0.99 for all studies (data not shown), indicating that alternative time windows within this range are unlikely to produce significant differences. Once evaporation flux stabilizes, extending the measurement period offers little additional value and increases the risk of concentration-related artifacts.

## Results

### Validation of Evaporimeter System—Pure Water

Evaporation of water from an aqueous-based solution is not a simple process. In addition to molecular properties, it depends on water concentration profiles in both the liquid and the surrounding vapor. The dissolution-diffusion mechanism for evaporation reduction of a solution compared to pure water evaporation has been previously detailed by Cerretani et al.^21^ The evaporative flux (µm/min) of pure water (*E*) is described as

(1) $$\begin{array}{c} E=\frac{1}{\rho_{w}\left(T_{s}\right)R_{m}}\left[\frac{P_{w}^{sat}\left(T_{s}\right)}{R_{g}T_{s}}-RH\frac{P_{w}^{sat}\left(T_{\infty }\right)}{R_{g}T_{\infty }}\right]\; \end{array}$$

where *R_m_* is the constant of environmental mass-transfer resistance (s/m) determined by airflow convection and temperature within the environmental chamber. $P_{w}^{sat}$ is the saturated vapor pressure of water at *T_s_*, the droplet surface temperature or at *T*_∞_, the environmental temperature, and ρ_*w*_(*T_s_*) is the molar density of water at *T_s_*. *RH* is the relative humidity, and *R_g_* is the ideal gas constant. Based on Equation (1), when measuring evaporative flux of water at a fixed temperature with various relative humidities, the evaporation flux should be linearly reduced at higher *RH*, provided that airflow profile in the chamber is unchanged.

**Figure 2.**
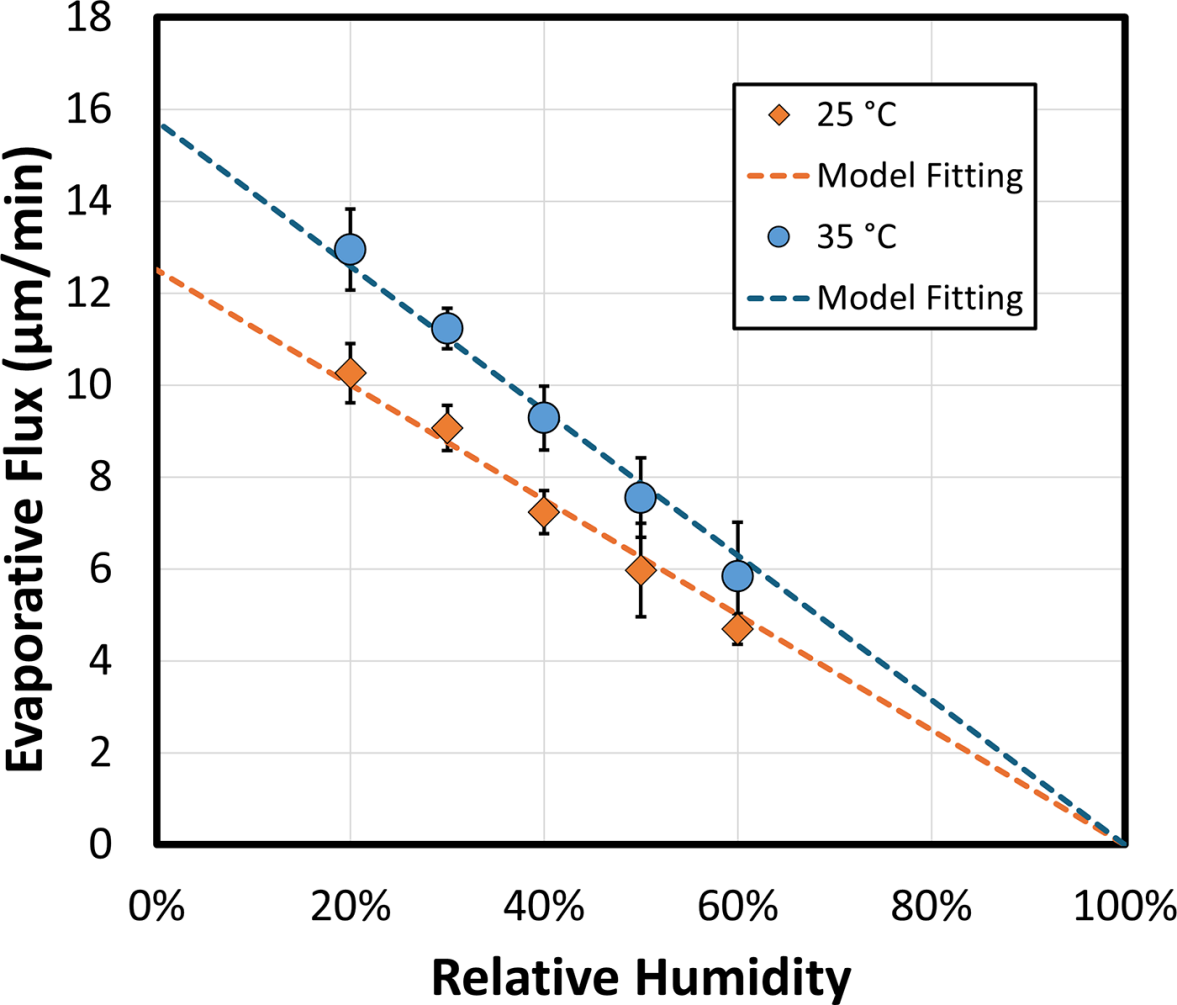
Water volumetric evaporative flux at different relative humidity at 25°C and 35°C, where *n* = 5 for each measurement condition. Data are plotted as mean ± SD. The *dashed lines* are the model-fitting results by Equation (1). The saturated vapor pressure is 3.1690 and 5.6267 kPa at 25°C and 35°C, respectively.^38^ The fitted constant of environmental mass-transfer resistance includes *R_m_* of 111 s/m at 25°C (*R*^2^ = 0.9984) and 152 s/m at 35°C (*R*^2^ = 0.9989), respectively.

Figure 2 shows the results of water evaporation measurement at various relative humidities ranging from 20% to 60% at 25°C (filled diamonds) and 35°C (filled circles). For simplicity, the decrease in surface temperature due to evaporation was neglected in the calculation by assuming *T_s_* = *T*_∞_. As indicated by the dashed lines in Figure 2, water evaporation at different temperatures and relative humidities aligns well with the kinetic mass-transfer model, demonstrating that the droplet-based evaporimeter provides stable kinetic flow conditions at fixed temperature, allowing accurate measurement of evaporative flux. The fitted mass-transfer resistances, *R_m_*, are 111 s/m at 25°C and 152 s/m at 35°C, respectively, suggesting that airflow in the environment chamber simulates conditions similar to conducting “sitting/reading” activities indoors.^22^^–^^24^ The small difference of the fitted *R_m_* between 25°C and 35°C indicates that natural convection airflow is not completely independent of temperature, likely due to small temperature gradient variations within the environment chamber. This can be further improved in the future by adding flow control inside the chamber to reduce any nonuniformities.

Additionally, the averaged surface tension measured during the evaporative rate measurement was 72.1 ± 0.8 mN/m at 25°C and 70.1 ± 0.9 mN/m at 35°C, which is consistent with literature values of 71.99 and 70.41 mN/m, respectively.^25^ This confirms the accuracy of the drop shape analysis from the system.

### Investigation of Evaporation Reduction Effect by Various Lubricating Eye Drops

To investigate the evaporation reduction of lubricating eye drop formulations, evaporation measurements were conducted at 35°C with a relative humidity of 40%. This condition was chosen to represent tear evaporation at ocular surface temperature in a typical indoor condition.^24^

**Figure 3.**
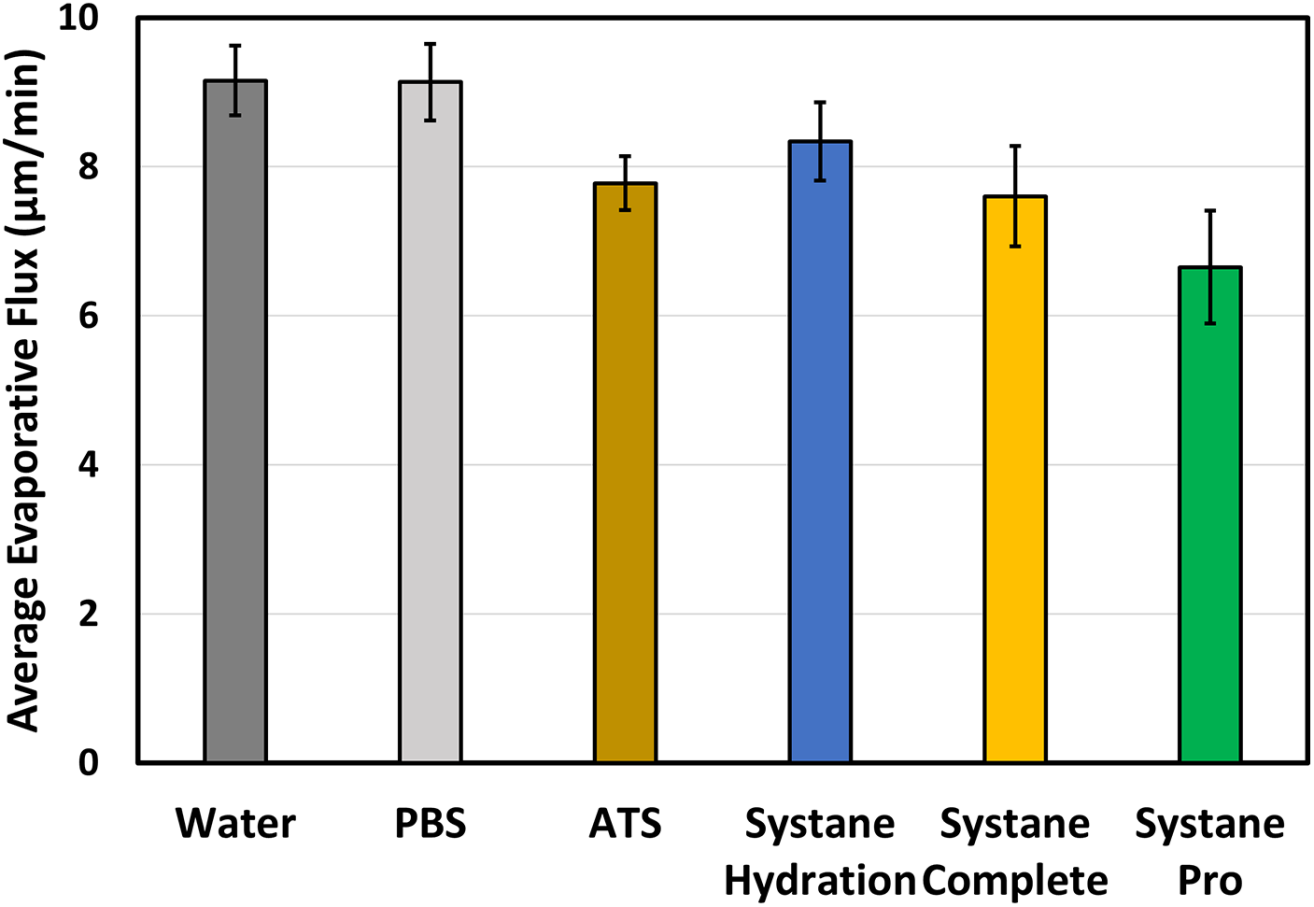
Averaged volumetric evaporative flux over 10 minutes at 35°C and relative humidity of 40% from baseline solutions and Systane eye drop formulations. Data are plotted as mean ± SD (*n* = 12 for water, *n* = 6 for PBS, *n* = 8 for Systane Complete, and *n* = 7 for other solutions).

Figure 3 presents the average volumetric evaporative flux results for three baseline solutions (i.e., water, PBS, and ATS) and three lubricating eye drop formulations (Systane Hydration, Systane Complete, and Systane Pro PF). PBS has a similar averaged evaporative flux compared to water (9.1 µm/min vs. 9.2 µm/min), consistent with the theory that salinity slightly lowers the saturated vapor pressure predicted by Raoult's law, potentially leading to a 1% to 2% reduction in evaporation.^26^^,^^27^ However, this reduction is not significant enough to be distinguished by the current evaporimeter study design. The simulated ATS, which contains multiple tear components (proteins, mucins, lipids) with osmolarity similar to normal tears or PBS, has a lower evaporative flux (7.8 µm/min) than both water and PBS. Systane Hydration, an eye drop containing 0.15% hyaluronic acid but no lipid additives, shows a slight reduction in evaporation compared to water (8.3 µm/min vs. 9.2 µm/min), but its averaged evaporative flux is higher than that of ATS. This aligns with the expectation that lipids provide better evaporation barriers than does hydrophilic hyaluronic acid.

Evaporation retardation is also observed in the evaporative flux results for Systane Complete, which contains mineral oil. The averaged evaporative flux is comparable between Systane Complete (7.6 µm/min) and ATS (7.8 µm/min). The combination of mineral oil and hyaluronic acid in Systane Pro PF, which contains both the same amount of mineral oil as Systane Complete and hyaluronic acid as Systane Hydration, demonstrates an additive and/or synergetic effect on evaporation reduction. There is a 28% lower evaporative flux for Systane Pro PF compared to that of water (6.7 µm/min vs. 9.2 µm/min).

Additional lubricating eye drop products containing oil and/or hyaluronic acid were investigated. The full results of measured evaporative flux over 10 minutes at 35°C are summarized in the Table. Most lubricating eye drops containing lipids have similar evaporative rates compared to ATS, in the range of 7.6 to 8.1 µm/min. Notably, while Blink Triple Care also contains both lipid (castor oil) and hyaluronic acid, it did not show a further evaporative reduction beyond the level of ATS, as did Systane Pro PF. This observation suggests that the potential synergetic effects between hyaluronic acid and lipid are influenced by the surrounding aqueous surfactants and emulsifiers and warrants further research.

**Table.** Measured Average Volumetric Evaporative Flux and the Standard Error of the Mean of Eye Drop Formulations over 10 Minutes at 35°C, Relative Humidity of 40%

Samples	Test Runs	Averaged Evaporative Flux (Mean ± SD, µm/min)	Standard Error of Mean (SD*/*$\sqrt{n}$)
Water	12	9.2 ± 0.5	0.13
PBS	6	9.1 ± 0.6	0.21
ATS	7	7.8 ± 0.4	0.14
Systane Hydration	7	8.3 ± 0.6	0.20
Systane Complete	8	7.6 ± 0.7	0.24
Systane Pro PF	7	6.7 ± 0.8	0.29
Blink Triple Care	7	8.1 ± 0.8	0.27
Refresh Optive Mega-3	7	8.1 ± 0.9	0.32
Refresh Optive Advanced	7	7.9 ± 0.8	0.27
iVIZIA	7	7.8 ± 0.7	0.23

## Discussion

**Figure 4.**
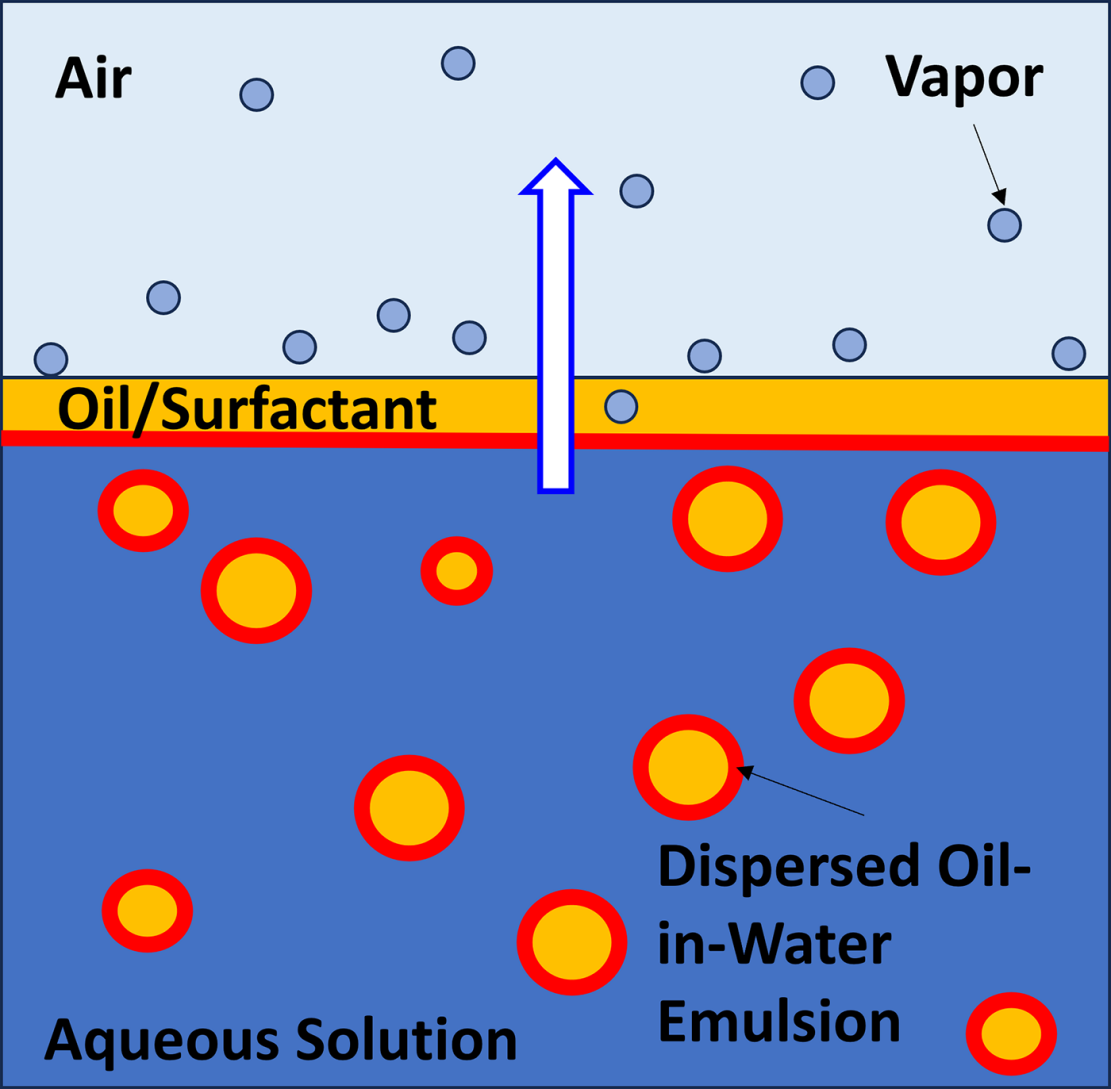
Schematic of the three-step evaporation process in oil-in-water emulsion lubricating eye drop formulation. *Orange* specifies lipid, which is coated with surfactant, illustrated in *red*. *Light blue* indicates evaporating water vapor molecules.

Lubricating eye drops typically contain ingredients such as salts to match tear osmolarity, along with additives such as wetting agents, lubricants, and sometimes preservatives and viscosity modifiers. Additionally, for formulations that include lipids, surfactant and emulsifiers are added to disperse the lipid as an oil-in-water emulsion.^28^ Each component and the complexity from their interactions, such as emulsifier concentration and emulsion droplet concentration and size, can influence the evaporation of lubricating eye drops. The schematic in Figure 4 pictures the physical process.^29^^,^^30^ Evaporation captured in Figure 4 involves multiple mass (water or vapor) transfer mechanisms in series. First, the emulsion accumulates near the nascent air/water interface. Depending on emulsion stability, some oil droplets may coalesce, forming a thin covering lipid layer.^31^^,^^32^ Water molecules transport through the creamed emulsion and dissolve into and diffuse through the oily layer, eventually escaping from the oil layer into the vapor phase. Clearly, when lipid is present in the eye drop formulation, repulsive forces between the surfactant-laden oil droplets (or surfactant micelles), their droplet size, and their interfacial tension all play important roles in emulsion stability and hence in the thickness of any protective lipid layer.

When no lipid is present in the eye drop solution, water molecules escape directly from the aqueous phase into the vapor phase. Equation (1) holds but with the vapor pressure at the surface now corresponding to the aqueous solution rather than to pure water. Thus, osmolytes in eye drop formulation can slow evaporation through lowering water vapor pressure. Nevertheless, environmental conditions, such as temperature, airflow velocity profiles, and relative humidity, dominate the overall process through the mass-transfer resistance *R_m_*.^21^^,^^24^^,^^33^ When an oily layer forms, Equation (1) remains valid. However, there are now additional series-protective resistances for water molecules to transverse across the lipid layer in addition to those in the surrounding air environment.^21^^,^^24^ These can be large depending on water solubility in the lipid and the layer thickness.

The described evaporation process is pertinent to tear evaporation through the tear film. In this context, the emulsified water phase in Figure 4 corresponds to the aqueous tear film, which contains various soluble tear components. The secondary lipid phase is the tear film lipid layer (TFLL), primarily composed of insoluble nonpolar lipids,^34^^,^^35^ and the closed evaporimeter chamber corresponds to the air environment exposed to the open eye. It is believed that TFLL lipids significantly contribute to reducing tear evaporation in a healthy eye, decreasing it by up to 90% compared to pure water evaporation at the same environmental conditions.^21^^,^^36^ Environmental factors are more relevant to managing chronic discomfort, especially if the individual's lifestyle involves constant exposure to harsh environmental conditions, such as dryness, heat, and wind.^5^

This study examines the evaporation rates of various lubricating eye drops. Systane Pro PF, an emulsion-based formulation containing both hyaluronic acid and mineral oil, demonstrated the greatest reduction in evaporative flux, suggesting a potential synergistic interaction between mineral oil and hyaluronic acid. Hyaluronic acid alone plays a minor role in reducing evaporation by slightly altering osmolarity and saturated vapor pressure,^37^ as evidenced by comparisons between the evaporative flux of Systane Hydration, water, and ATS. Thus, the synergistic effect is hypothesized to mainly arise from hyaluronic acid facilitating the formation of a more efficient diffusion barrier arising from a mineral oil lipid layer blanketing the aqueous surface. Further investigation into the interactions between hyaluronic acid and mineral oil, as well as their interfacial behavior with air and water, is necessary to elucidate this mechanism.

Evaporation of the emulsified solution is further influenced by the complexity of the emulsion. The stability of the oil-in-water emulsion, determined by the intricate balance between surfactant, water, oil, and solutes, governs the formation rate of a creaming layer near the air/water interface due to density differences between the oil and water phases, as well as the rate of coalescence and eventual collapse of the emulsion, leading to phase separation of the insoluble oil into the aqueous/air interface. If no emulsion coalescence occurs into a spread oily layer, little additional reduction of evaporation is expected upon adding lipid. Thus, control of emulsion stability is important in lipid-containing eye care solutions. A well-controlled study on the formulation is required to gain deeper insight into these complexities.

Overall, we observed a much smaller evaporation reduction from these lubricating eye drop formulations compared to earlier studies, particularly for lipid-containing eye drop emulsions. For example, Lin and Svitova^18^ reported a 43% relative evaporative reduction of Refresh Optive Mega-3 compared to that of their model tear electrolyte solutions, a much stronger evaporation retardation compared to the 11% reduction observed with PBS in this study. While both approaches utilize the droplet evaporimeter technique, the methodologies and sample conditions differ. In particular, sample concentration is a critical factor influencing measurement outcomes. Here, we focus on measuring the initial evaporation flux within 10 minutes immediately after dispensing the eye drop formulation as a fast-screening method for lubricating eye drop formulation development. Conversely, Svitova and Lin^15^ and Lin and Svitova^18^ conducted their evaporation measurement after an extended “preequilibrium” period of up to 48 hours. It is expected that after prolonged exposure to open air, the formulations, especially the emulsion-based ones, might gradually become more unstable, eventually leading to creaming and/or a complete phase separation of the oil-in-water emulsion into a thick oily film. These possible events increase the water molecular diffusion resistance at the water/air interface by increasing the oily layer thickness previously described in Figure 4 and thus further reducing the evaporative flux.

In this study, we present an advanced evaporimeter system for characterizing eye drop evaporation dynamics that establishes a scientific framework for droplet-based in vitro measurements—a method previously underexplored despite its sensitivity to experimental and sample conditions. We analyze key factors influencing evaporation, including environmental control, instrument setup, sample properties, and kinetic regulation, addressing a critical knowledge gap. Additionally, we propose a standardized protocol that ensures reliable, reproducible measurements within practical time frames, providing a robust foundation for future research and ophthalmic formulation development.

## Conclusions

A droplet-based evaporimeter system was developed by modifying a goniometer and integrating an environmental control unit, creating a research tool to investigate the evaporation of lubricating eye drop formulations and potentially other ophthalmic applications. This modified evaporimeter allows precise measurement of evaporation rates under varying temperature and humidity conditions, as validated through evaporation experiments with water and through consistency with modeled mass-transfer kinetics. Designed as a rapid screening tool for eye drop formulations, the apparatus effectively measures the evaporative flux, as demonstrated by the results from multiple commercial ophthalmic lubricating eye drops. Beyond formulation screening, the system will be utilized further to explore how interactions between eye drop formulations and tear film components influence evaporation, both in vitro and ex vivo, with the goal of advancing treatments for dry eye relief.

## References

[1] Willcox MDP, Argüeso P, Georgiev GA, et al. TFOS DEWS II tear film report. *Ocul Surf*. 2017; 15(3): 366–403. 28736338 10.1016/j.jtos.2017.03.006 PMC6035753

[2] Craig J . Structure and function of the preocular tear film. In: Edwards R, Hutchins C, eds. *The Tear Film: Structure, Function and Clinical Examination*. Vol. 1. Boston, MA: Butterworth-Heinemann Oxford; 2002: 18–50.

[3] Dilly PN . Structure and function of the tear film. In: Sullivan DA, ed. *Lacrimal Gland, Tear Film, and Dry Eye Syndromes: Basic Science and Clinical Relevance*. New York, NY, USA: Springer US; 1994: 239–247.

[4] Craig JP, Nichols KK, Akpek EK, et al. TFOS DEWS II definition and classification report. *Ocul Surf*. 2017; 15(3): 276–283. 28736335 10.1016/j.jtos.2017.05.008

[5] Jones L, Downie LE, Korb D, et al. TFOS DEWS II management and therapy report. *Ocul Surf*. 2017; 15(3): 575–628. 28736343 10.1016/j.jtos.2017.05.006

[6] Wong S, Murphy PJ, Jones L. Tear evaporation rates: what does the literature tell us? *Contact Lens Anterior Eye*. 2018; 41(3): 297–306. 29277480 10.1016/j.clae.2017.12.003

[7] Mathers W . Evaporation from the ocular surface. *Exp Eye Res*. 2004; 78(3): 389–394. 15106917 10.1016/s0014-4835(03)00199-4

[8] Tan JH, Ng EYK, Acharya UR. Evaluation of tear evaporation from ocular surface by functional infrared thermography. *Med Phys*. 2010; 37(11): 6022–6034. 21158314 10.1118/1.3495540

[9] Petznick A, Tan JH, Boo SK, Lee SY, Acharya UR, Tong L. Repeatability of a new method for measuring tear evaporation rates. *Optom Vis Sci*. 2013; 90(4): 366–371. 23435224 10.1097/OPX.0b013e318288bdd1

[10] Yeo S, Tan JH, Acharya UR, Sudarshan VK, Tong L. Longitudinal changes in tear evaporation rates after eyelid warming therapies in meibomian gland dysfunction. *Invest Ophthalmol Vis Sci*. 2016; 57(4): 1974–1981. 27096755 10.1167/iovs.16-19088

[11] Nichols JJ, Mitchell GL, King-Smith PE. Thinning rate of the precorneal and prelens tear films. *Invest Ophthalmol Vis Sci*. 2005; 46(7): 2353–2361. 15980222 10.1167/iovs.05-0094

[12] Kimball SH, King-Smith PE, Nichols JJ. Evidence for the major contribution of evaporation to tear film thinning between blinks. *Invest Ophthalmol Vis Sci*. 2010; 51(12): 6294–6297. 20688724 10.1167/iovs.09-4772 PMC3055755

[13] Nichols JJ, King-Smith PE, Hinel EA, Thangavelu M, Nichols KK. The use of fluorescent quenching in studying the contribution of evaporation to tear thinning. *Invest Ophthalmol Vis Sci*. 2012; 53(9): 5426–5432. 22789918 10.1167/iovs.12-10033 PMC3423665

[14] Bai M, Holeva KT. Compositions providing improved eye comfort. US Patent Application no 16/113,076, Published online 2019.

[15] Svitova TF, Lin MC. Evaporation retardation by model tear-lipid films: the roles of film aging, compositions and interfacial rheological properties. *Colloids Surfaces B Biointerfaces*. 2021; 197: 111392. 33189036 10.1016/j.colsurfb.2020.111392

[16] Miano F, Calcara M, Giuliano F, Millar TJ, Enea V. Effect of meibomian lipid layer on evaporation of tears. *J Phys Condens Matter*. 2004; 16(26): S2461.

[17] Xu X, Li G, Zuo YY. Effect of model tear film lipid layer on water evaporation. *Invest Ophthalmol Vis Sci*. 2023; 64(1): 13. 10.1167/iovs.64.1.13 PMC9872843 36656568

[18] Lin MC, Svitova TF. Effects of model tear proteins and topical ophthalmic formulations on evaporation inhibition and biophysical property of model tear lipid nanofilm in vitro. *JCIS Open*. 2021; 4: 100028.

[19] Lin MC, Svitova T, Ding J. Impact of whole tear aqueous components on evaporation through human tear lipid films in vitro. *Invest Ophthalmol Vis Sci*. 2024; 65(7): 6554.

[20] Phan CM, Chan VWY, Drolle E, et al. Evaluating the in vitro wettability and coefficient of friction of a novel and contemporary reusable silicone hydrogel contact lens materials using an in vitro blink model. *Contact Lens Anterior Eye*. 2024; 47(2): 102129. 38423868 10.1016/j.clae.2024.102129

[21] Cerretani CF, Ho NH, Radke CJ. Water-evaporation reduction by duplex films: application to the human tear film. *Adv Colloid Interface Sci*. 2013; 197: 33–57. 23694847 10.1016/j.cis.2013.03.007

[22] Baldwin PEJ, Maynard AD. A survey of wind speeds in indoor workplaces. *Ann Occup Hyg*. 1998; 42(5): 303–313. 9729918 10.1016/s0003-4878(98)00031-3

[23] Fornasiero F, Prausnitz JM, Radke CJ. Post-lens tear-film depletion due to evaporative dehydration of a soft contact lens. *J Memb Sci*. 2006; 275(1): 229–243.

[24] Peng CC, Cerretani C, Braun RJ, Radke CJ. Evaporation-driven instability of the precorneal tear film. *Adv Colloid Interface Sci*. 2014; 206: 250–264. 23842140 10.1016/j.cis.2013.06.001

[25] Vargaftik NB, Volkov BN, Voljak LD. International tables of the surface tension of water. *J Phys Chem Ref Data*. 1983; 12(3): 817–820.

[26] Al-Shammiri M . Evaporation rate as a function of water salinity. *Desalination*. 2002; 150(2): 189–203.

[27] Salhotra AM, Adams EE, Harleman DRF. Effect of salinity and ionic composition on evaporation: analysis of Dead Sea evaporation pans. *Water Resour Res*. 1985; 21(9): 1336–1344.

[28] Lee SY, Tong L. Lipid-containing lubricants for dry eye: a systematic review. *Optom Vis Sci*. 2012; 89(11): 1654–1661. 23096494 10.1097/OPX.0b013e31826f32e0

[29] Gutiérrez G, Benito JM, Coca J, Pazos C. Vacuum evaporation of surfactant solutions and oil-in-water emulsions. *Chem Eng J*. 2010; 162(1): 201–207.

[30] Li KY, Tu H, Ray AK. Charge limits on droplets during evaporation. *Langmuir*. 2005; 21(9): 3786–3794. 15835938 10.1021/la047973n

[31] Aranberri I, Binks BP, Clint JH, Fletcher PDI. Evaporation rates of water from concentrated oil-in-water emulsions. *Langmuir*. 2004; 20(6): 2069–2074. 15835653 10.1021/la035031x

[32] Aranberri I, Beverley KJ, Binks BP, Clint JH, Fletcher PDI. How do emulsions evaporate? *Langmuir*. 2002; 18(9): 3471–3475.

[33] Peng CC, Cerretani C, Li Y, et al. Flow evaporimeter to assess evaporative resistance of human tear-film lipid layer. *Ind Eng Chem Res*. 2014; 53(47): 18130–18139.

[34] Butovich IA . Tear film lipids. *Exp Eye Res*. 2013; 117: 4–27. 23769846 10.1016/j.exer.2013.05.010 PMC3844095

[35] Rantamäki AH, Seppänen-Laakso T, Oresic M, Jauhiainen M, Holopainen JM. Human tear fluid lipidome: from composition to function. *PLoS One*. 2011; 6(5): 1–7. 10.1371/journal.pone.0019553 PMC3088682 21573170

[36] Mishima S, Maurice DM. The oily layer of the tear film and evaporation from the corneal surface. *Exp Eye Res*. 1961; 1(1): 39–45. 14474548 10.1016/s0014-4835(61)80006-7

[37] Borkar S, Baumli P, Vance T, et al. Elucidating the roles of electrolytes and hydrogen bonding in the dewetting dynamics of the tear film. *Proc Natl Acad Sci*. 2024; 121(31): e2407501121. 39042697 10.1073/pnas.2407501121 PMC11295001

[38] Lide DR , ed. Section 6: Fluid properties. In: Lide David R. *CRC Handbook of Chemistry and Physics*. 85th ed. Boca Raton, FL: CRC Press; 2004: 6–10.

